# Altered resting state brain metabolic connectivity in dementia with Lewy bodies

**DOI:** 10.3389/fneur.2022.847935

**Published:** 2022-08-08

**Authors:** Euna Choi, Ji Won Han, Seung Wan Suh, Jong Bin Bae, Ji Hyun Han, Subin Lee, Sang Eun Kim, Ki Woong Kim

**Affiliations:** ^1^Department of Brain and Cognitive Science, Seoul National University College of Natural Sciences, Seoul, South Korea; ^2^Department of Neuropsychiatry, Seoul National University Bundang Hospital, Seongnam, South Korea; ^3^Department of Psychiatry, Kangdong Sacred Heart Hospital, Hallym University College of Medicine, Seoul, South Korea; ^4^Department of Nuclear Medicine, Seoul National University Bundang Hospital, Seongnam, South Korea; ^5^Department of Nuclear Medicine, Seoul National University College of Medicine, Seoul, South Korea; ^6^Center for Nanomolecular Imaging and Innovative Drug Development, Advanced Institutes of Convergence Technology, Suwon, South Korea; ^7^Department of Psychiatry, Seoul National University College of Medicine, Seoul, South Korea

**Keywords:** dementia with Lewy bodies, Parkinsonism, metabolic connectivity, positron emission tomography, neurodegenerative disease

## Abstract

Although dementia with Lewy bodies (DLB) have Parkinsonism in common with Parkinson's disease (PD) or PD dementia (PDD), they have different neuropathologies that underlie Parkinsonism. Altered brain functional connectivity that may correspond to neuropathology has been reported in PD while never been studied in DLB. To identify the characteristic brain connectivity of Parkinsonism in DLB, we compared the resting state metabolic connectivity in striato-thalamo-cortical (STC) circuit, nigrostriatal pathway, and cerebello-thalamo-cortical motor (CTC) circuit in 27 patients with drug-naïve DLB and 27 age- and sex-matched normal controls using 18F-fluoro-2-deoxyglucose PET. We derived 118 regions of interest using the Automated Anatomical Labeling templates and the Wake Forest University Pick-Atlas. We applied the sparse inverse covariance estimation method to construct the metabolic connectivity matrix. Patients with DLB, with or without Parkinsonism, showed lower inter-regional connectivity between the areas included in the STC circuit (motor cortex–striatum, midbrain–striatum, striatum–globus pallidus, and globus pallidus–thalamus) than the controls. DLB patients with Parkinsonism showed less reduced inter-regional connectivity between the midbrain and the striatum than those without Parkinsonism, and higher inter-regional connectivity between the areas included in the CTC circuit (motor cortex–pons, pons–cerebellum, and cerebellum–thalamus) than those without Parkinsonism and the controls. The resting state metabolic connectivity in the STC circuit may be reduced in DLB. In DLB with Parkinsonism, the CTC circuit and the nigrostriatal pathway may be activated to mitigate Parkinsonism. This difference in the brain connectivity may be a candidate biomarker for differentiating DLB from PD or PDD.

## Introduction

Dementia with Lewy bodies (DLB) and Parkinson's disease dementia (PDD) together represent the second most common cause of dementia (1). About 30% of patients with Parkinson's disease (PD) have cognitive symptoms at initial diagnosis and as many as 80% will develop cognitive symptoms at some point in their disease (2). About 25–50% of patients with DLB show Parkinsonism at initial diagnosis and as many as 80% eventually develop Parkinsonism as the disease progresses (3). Since PDD commonly shows core diagnostic features of DLB, it can be diagnosed when Parkinsonian motor symptoms start at least 1 year earlier than cognitive or perceptual symptoms. In DLB, cognitive symptoms appear before, or at the same time, as motor symptoms (4). However, this 1-year rule has logical and practical limitations. For example, it is impossible to distinguish PD from DLB on the 1-year rule when they are prodromal. It is also difficult to determine whether PD with mild cognitive impairment (MCI) would be PDD or DLB (5).

Although PDD and DLB have Parkinsonism in common, DLB shows more rigidity and bradykinesia than resting tremors and shows the symptoms more symmetrically than dose PDD (6). Although both diseases have Lewy pathology in common, it takes place from the brainstem in PD (7) while from the neocortex or limbic system in DLB (4). Therefore, we may differentiate PD or PDD from DLB on this different motor pathology even when non-motor symptoms such as cognitive impairment or hallucination are not accompanied.

In PD, nigrostriatal dopaminergic degeneration may produce rigidity and bradykinesia by impairing the striato-thalamo-cortical (STC) motor circuit (8). In previous resting state functional MRI (fMRI) studies, PD showed reduced connectivity between the substantia nigra and the putamen (9) while enhanced connectivity between the motor cortex and the striatum (10, 11) and between the motor cortex and the cerebellum (12). Since the severity of Parkinsonism was positively correlated with the connectivity of the cerebello-thalamo-cortical (CTC) motor circuit (12), the cerebellum and possibly the motor cortex may play a compensatory role to maintain a better motor function in PD (13).

Since DLB has more Lewy pathology in the striatum and the neocortex (4, 14), DLB may have different resting state brain connectivity patterns in STC motor circuit from PD. However, the resting state brain connectivity in STC motor circuit has never been investigated in DLB. The resting-state brain connectivity can be obtained using the temporal coherence of BOLD signals between different brain regions measured with fMRI during the resting state as in the PD study presented above. It can also be obtained through the covariation of the ^18^F-fluoro-2-deoxyglucose PET (FDG-PET) glucose uptake value between the brain regions of the subjects, which is called metabolic connectivity. Brain metabolic connectivity measured through FDG-PET data examines functional interactions in brain regions and can provide valuable insights into understanding the pathophysiology of neurodegenerative disease (15). In this study, we investigated the connectivity of STC and CTC circuits and nigrostriatal pathway in DLB by comparing the resting state metabolic connectivity between patients with DLB and their age- and sex-matched cognitively normal controls (NC) using FDG-PET.

## Materials and methods

### Subjects

We enrolled 27 patients with DLB from visitors to the dementia clinic of Seoul National University Bundang Hospital (SNUBH) from 2006 to 2017 and their 27 age- and sex-matched cognitively NC from visitors to the dementia clinic of SNUBH from 2006 to 2017 and the participants of the Korean Longitudinal Study on Cognitive Aging and Dementia (KLOSCAD). The KLOSCAD is an ongoing nationwide population-based prospective cohort study on cognitive aging and dementia of elderly Koreans launched in 2009 (16). All subjects were community-dwelling Koreans aged 60 years or older. All patients with DLB were naïve from anti-Parkinsonian or antipsychotic medications and did not take any antidepressants or prokinetics known to act on dopamine receptors. In addition, they had no other comorbid major psychiatric or neurologic diseases. We summarized the characteristics of the study participants in Table 1.

**Table 1:** Characteristics of the participants.

	***NC^*a*^ (N = 27)***	***DLB***			***p (post-hoc)***		
		**All (*N* = 27)**	**P-DLB^b^ (*N* = 17)**	**P+DLB^c^ (*N* = 10)**	**NC vs. All DLB**	**NC vs. P-DLB vs. P+DLB**	**P-DLB vs. P+DLB**
Age (years, mean ± SD)	73.3 ± 5.3	72.7 ± 5.2	71.6 ± 4.5	74.7 ± 5.9	1.000	0.579	-
Sex (Women, %)	63.0	63.0	70.6	50.0	0.680	0.564	-
Education (years, mean ± SD)	12.6 ± 4.4	11.2 ± 5.4	11.1 ± 4.8	11.5 ± 6.6	0.298	0.571	-
MMSE (scores, mean ± SD)	26.3 ± 2.3	21.1 ± 5.1	22.5 ± 4.8	18.6 ± 4.7	<0.001	<0.001 (a>b***, b>c*, a>c***)	-
Disease duration (year, mean ± SD)	-	1.8± 2.2	2.1 ± 2.7	1.2 ± 0.9	-	-	0.882
Visual hallucination (+, %)	-	51.9	52.9	50	-	-	0.253
UPDRS-III(scores, mean ± SD, range)	-	-	-	21.1 ± 12.9 (4–39)	-	-	-

NC, Normal controls; DLB, dementia with Lewy bodies; P-DLB, dementia with Lewy bodies without Parkinsonism; P+DLB, dementia with Lewy bodies with Parkinsonism; MMSE, Mini-Mental State Examination; SD, Standard deviation. Chi-squared tests for categorical variables and independent t-test or analysis of variance for continuous variables. Bonferroni's post-hoc test was performed for three group comparisons (a = NC; b = P-DLB; c = P+DLB,

^*^p < 0.05,

^***^p < 0.001).

Geriatric psychiatrists, with expertise in dementia research, administered a standard diagnostic face-to-face interview, including recording detailed medical histories and conducting physical and neurological examination of each participant, using the Korean version of the Consortium to Establish a Registry for Alzheimer's Disease Assessment Packet (CERAD-K) Clinical Assessment Battery (17) and the Korean version of Mini International Neuropsychiatric Interview (MINI) (18). They evaluated Parkinsonian symptoms using the Extrapyramidal Dysfunction in AD (EPDAD) scale included in the CERAD-K (19). A research neuropsychologist or trained research nurse conducted neuropsychological assessments, including the CERAD-K Neuropsychological Assessment Battery (17), the Digit Span Test (20), and the frontal assessment battery (FAB) (21). We also conducted laboratory tests, including complete blood cell counts, chemical profiles, serologic tests for syphilis, and typing of apolipoprotein E genes. We diagnosed DLB with the revised consensus criteria proposed by McKeith et al. (4). Among the 27 patients with DLB, 10 either had rigidity or bradykinesia, but no tremor (P+DLB group), but 17 did not have any of the above symptoms (P-DLB group).

### Image acquisition and preprocessing

We acquired brain ^18^F-FDG-PET images at the diagnosis from a dedicated PET scanner (Allegro; Philips Medical System, Cleveland, OH, USA) (22) in 54 participants (27 DLB and 27 NC). The amount of intravenous administration of ^18^F-FDG was 4.8 MBq/kg for the PET scanner. We instructed the participants to fast for at least 6 h before scanning. After the fasting period, we checked that the blood sugar level of the participants was below 180 mg/dl. We then injected ^18^F-FDG intravenously in a quiet, dimly lit waiting room, and allowed the participants to lie comfortably for a 40-min FDG equilibration period. After the equilibrium period, we led the participants to the adjacent imaging suite and aligned their head to the canthomeatal line in the scanner. From the Allegro PET scanner, we obtained 10-min emission scans and attenuation maps, using a Cs-137 transmission source. We reconstructed the attenuation-corrected images using the PET data and a 3D row-action maximum-likelihood algorithm with a 3D image filter of 128 × 128 × 90 matrices and a pixel size of 2 × 2 × 2 mm.

We preprocessed the image data using the Statistical Parametric Mapping 8 (SPM8) software (www.fil.ion.ucl.ac.uk/spm/) based on MATLAB 2014a (www.mathworks.com). We aligned the FDG-PET images of each subject to standard Montreal Neurological Institute (MNI) space by spatial normalization and resampled them to a 2-mm isovoxel resolution (23). We smoothed all the spatially normalized PET images using a Gaussian kernel of 8-mm full width at half maximum (FWHM) to increase the signal-to-noise ratio and to minimize the individual differences in the uncorrected brain cortex. We derived 116 regions of interest (ROIs) consisting of 90 cortical and subcortical regions and 26 cerebellar regions, using the Automated Anatomical Labeling (AAL) templates (24) and 2 ROIs consisting of the brainstem, using the Wake Forest University PickAtlas Tailarach Daemon (25). We calculated the regional standard uptake value ratio (SUVR) of each ROI by normalizing to activity in mean values of the bilateral postcentral gyrus as a reference region because the postcentral area is a relatively preserved area in DLB (26).

**Figure 1:**
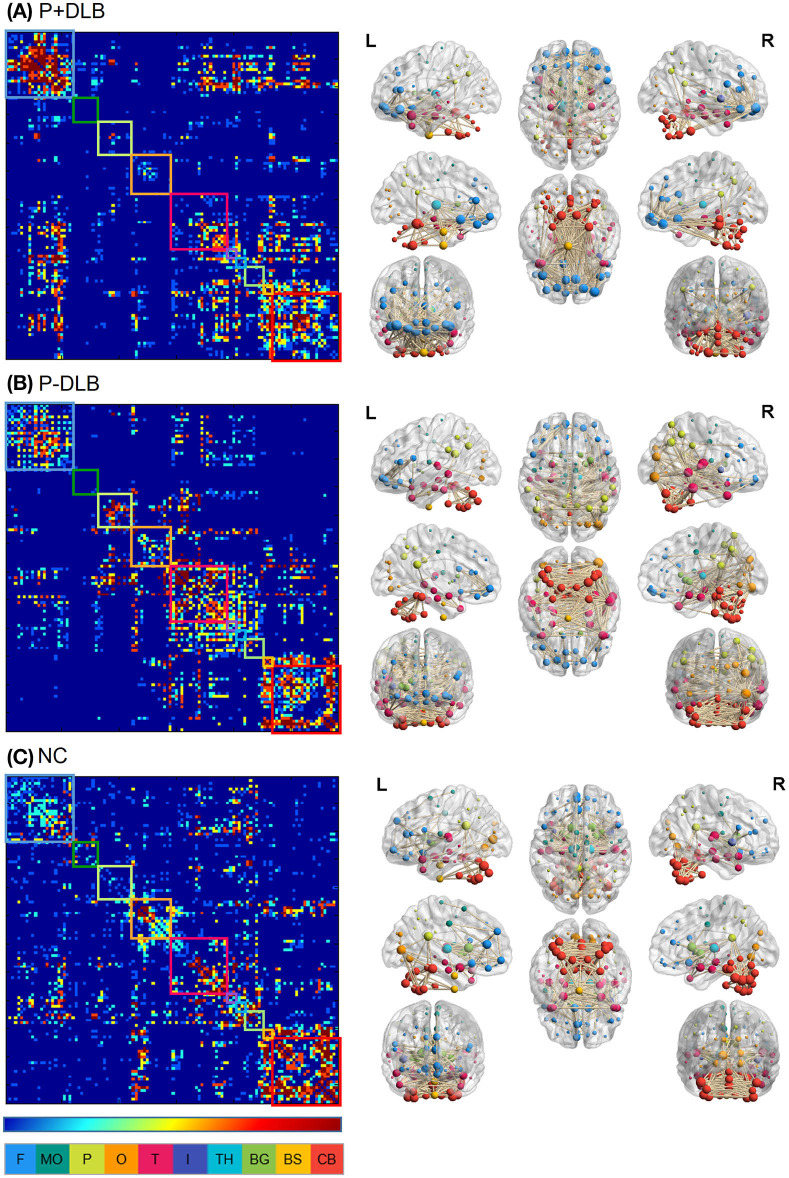
Intra- and inter-regional connectivity matrices of the **(A)** P+DLB, **(B)** P-DLB, and **(C)** NC groups. Full connectivity matrices in the left column were obtained from 116 nodes. The color bar represents the strength of connectivity, with red representing stronger connectivity, and blue representing weaker connectivity. Each box represents the 10 subdivisions, which are F, MC, P, O, T, I, TH, BG, BS, and CB. The color of each subdivision matches the color of the node in the brain template in the right column. We display connectivity matrices on 3D brain templates on the right column. P+DLB, dementia with Lewy bodies with parkinsonism; P-DLB, dementia with Lewy bodies without parkinsonism; NC, normal controls; F, frontal; MC, motor cortex; P, parietal; O, occipital; T, temporal; I, insular; TH, thalamus; BG, basal ganglia; BS, brainstem; CB, cerebellum.

We defined the 116 ROIs identified above as the nodes and made up the network, with the frontal, motor cortex, parietal, occipital, temporal, insular, thalamus, basal ganglia, brainstem, and cerebellum as 10 subdivisions (Figure 1). We excluded the bilateral postcentral gyrus, which was used as the reference region. To analyze the connectivity of the STC and CTC circuits, we estimated the resting state metabolic connectivity between seven subdivisions: including the motor cortex, striatum, globus pallidus, midbrain, thalamus, pons, and cerebellum. We constructed a subject-by-node matrix (number of subjects × 116 regions) from each diagnostic group using the SUVR. We applied the sparse inverse covariance estimation (SICE) method to the subject-by-node matrices (27) using the GraphVar toolbox (28) to construct the metabolic connectivity matrix, in which 0 and 1 represent the absence and the presence of connections (significant partial correlation between two nodes) at a specific density, respectively. The SICE method we used to estimate metabolic connectivity has been previously used in other FDG-PET studies (29, 30) because the brain connectivity model can be reliably provided by SICE even if the sample size is equal or even smaller than the number of nodes selected (27). Since SICE does not provide information on strength of metabolic connectivity, we obtained a quasi-measure of the strength by summing the unweighted binary matrices estimated at several different density levels (0.03, 0.05, 0.07, 0.09, 0.12) (27) for visualization purposes (Figure 1L). We visualized the metabolic connectivity matrix on a 3D brain template using the BrainNet toolbox (31) (Figure 1R).

### Statistical analysis

We compared the characteristics between diagnostic groups using the chi-squared tests for categorical variables and the independent *t*-tests or ANOVA for continuous variables.

To compare the brain connectivity in each group, we calculated the number of connections at a specific threshold (0.03) (27). The number of connections was referred to the number of nodes marked 1 in the metabolic connection matrix. We defined the number of connections between the different subdivisions as inter-regional connectivity and the number of connections in each subdivision as intra-regional connectivity. For statistical analysis, we extracted 1,000 bootstrap samples from each subject group, with replacement. For each of the bootstrap samples, we calculated the number of connections in intra- and inter-subdivisions, as described above (27). We compared the inter-regional connectivities (motor cortex–striatum, midbrain–striatum, striatum–globus pallidus, globus pallidus–thalamus, thalamus–motor cortex, motor cortex–pons, pons–cerebellum, and cerebellum–thalamus) between P+DLB, P-DLB, and NC groups using ANOVA with the Bonferroni *post-hoc* comparisons. We also compared the intra-regional connectivities (motor cortex, midbrain, striatum, globus pallidus, thalamus, pons, and cerebellum) between P+DLB, P-DLB, and NC groups using ANOVA with the Bonferroni *post-hoc* comparisons. We performed all statistical analysis using the IBM SPSS version 20 (32).

## Result

The intra- and inter-regional connectivity matrices of the P+DLB, P-DLB and NC groups are shown in Figure 1. The inter-regional connectivity in the STC and CTC circuits was different between the NC, P+DLB, and P-DLB groups; motor cortex–striatum connectivity (F_2, 2997_ = 1,021.02, *p* < 0.001, η^2^ = 0.405), midbrain–striatum connectivity (F_2, 2997_ = 153.91, *p* < 0.001, η^2^ = 0.093), striatum–globus pallidus connectivity (F_2, 2997_ = 7210.17, *p* < 0.001, η^2^ = 0.828), globus pallidus–thalamus connectivity (F_2, 2997_ = 989.52, *p* < 0.001, η^2^ = 0.398), thalamus–motor cortex connectivity (F_2, 2997_ = 314.80, *p* < 0.001, η^2^ = 0.174), motor cortex–pons connectivity (F_2, 2997_ = 7.04, *p* < 0.001, η^2^ = 0.005), pons–cerebellum connectivity (F_2, 2997_ = 148.81, *p* < 0.001, η^2^ = 0.090), and cerebellum–thalamus connectivity (F_2, 2997_ = 1,248.15, *p* < 0.001, η^2^ = 0.454).

**Figure 2:**
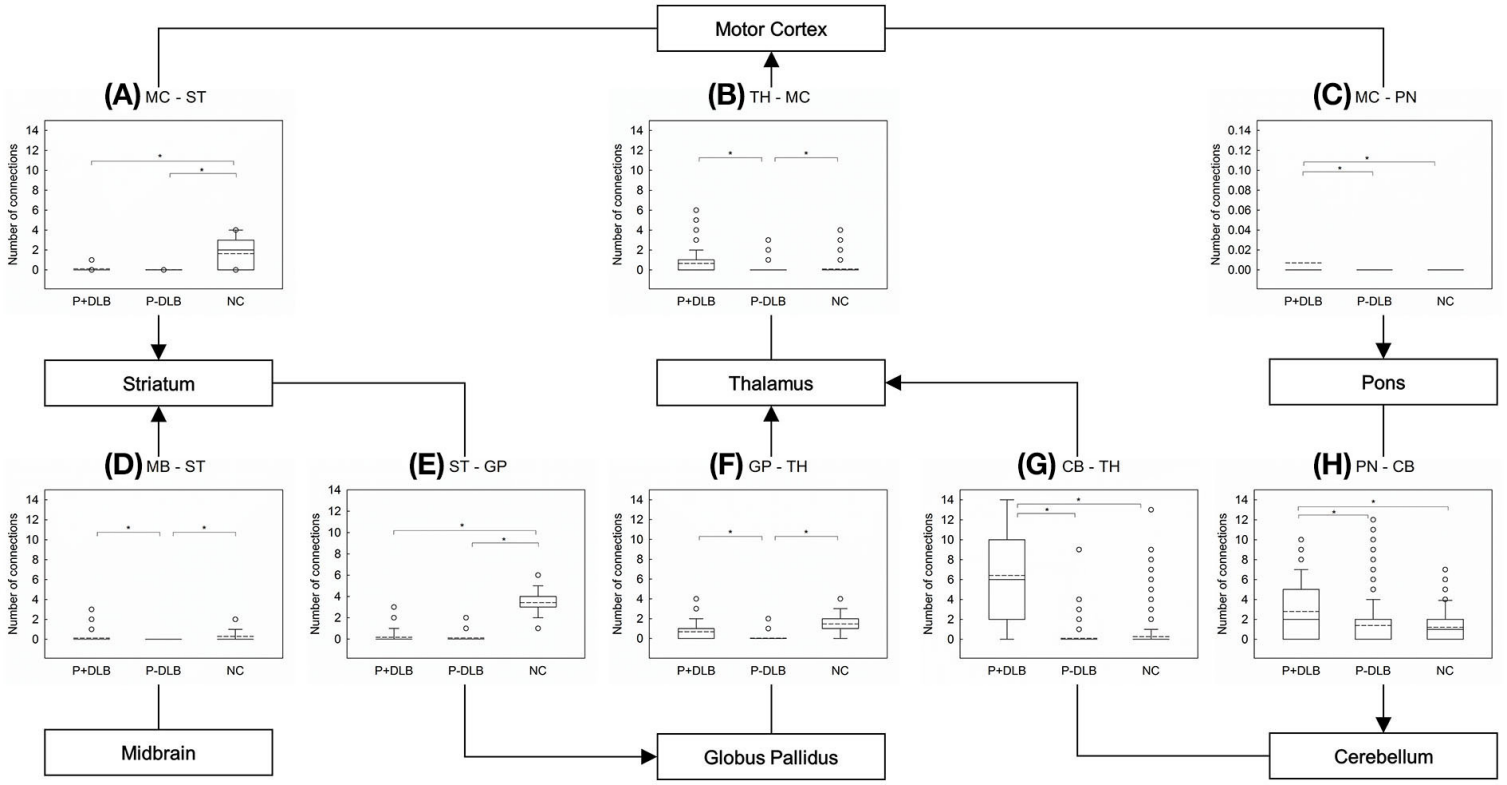
Comparison of the number of inter-regional connections among P+DLB, P-DLB, and NC groups. Box plots of the mean number of connections between **(A)** MC–ST; **(B)** TH-MC, **(C)** MC-PN, **(D)** MB-ST, **(E)** ST-GP, **(F)** GP-TH, **(G)** CB-TH, and **(H)** PN-CB. The solid lines represent the median value and dashed lines represent the mean value. *indicate significantly different group means at *p* <0.001. P+DLB, dementia with Lewy bodies with parkinsonism; P-DLB, dementia with Lewy bodies without parkinsonism; NC, normal controls; MC, motor cortex; ST, striatum; PN, pons; TH, thalamus; MB, midbrain; GP, globus pallidus; CB, cerebellum.

*Post-hoc* comparisons showed that the inter-regional connectivities within the STC circuit were lower in both P+DLB and P-DLB than in NC. The motor cortex–striatum and striatum–globus pallidus connectivities of P+DLB were comparable to those of P-DLB (Figures 2A,E), while the globus pallidus–thalamus and midbrain–striatum connectivities of P+DLB were higher than those of P-DLB (Figures 2D,F). The inter-regional connectivities within the CTC circuit (motor cortex–pons, pons–cerebellum, and cerebellum–thalamus connectivities) of P+DLB were higher than those of P-DLB, as well as those of NC. P-DLB showed comparable connectivity between the motor cortex, pons, cerebellum, and thalamus to NC (Figures 2C,G,H). The thalamus–motor cortex connectivity, which is shared by the STC and CTC circuits, was highest in P+DLB, followed by NC and P-DLB (Figure 2B).

**Figure 3:**
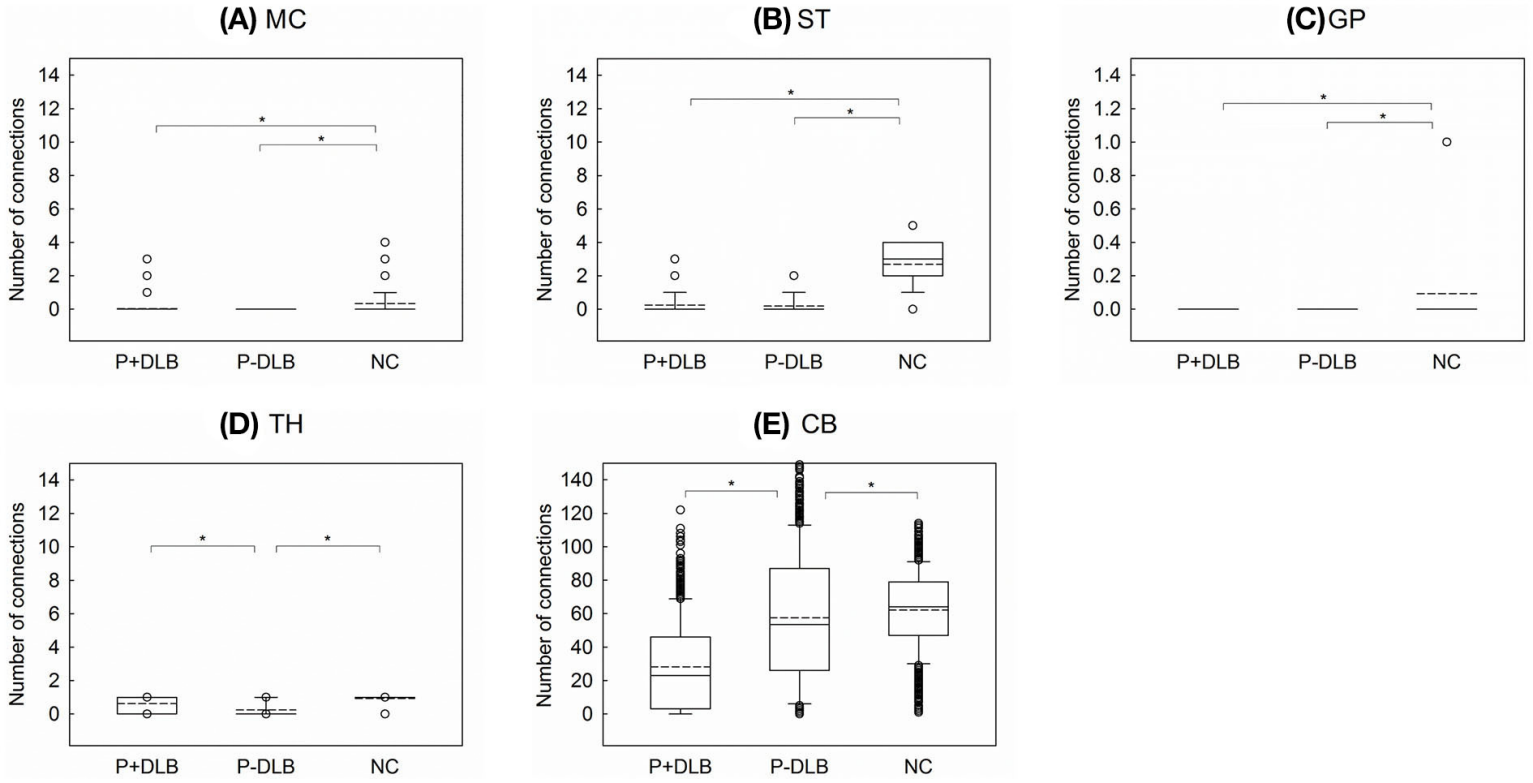
Comparison of the number of intra-regional connections among P+DLB, P-DLB, and NC groups. Box plots of the mean number of connections between **(A)** MC, **(B)** ST, **(C)** GP, **(D)** TH, and **(E)** CB. The solid lines represent the median value and the dashed lines represent the mean value. *indicate significantly different group means at *p* <0.001. P+DLB, dementia with Lewy bodies with parkinsonism; P-DLB, dementia with Lewy bodies without parkinsonism; NC, normal controls; MC, motor cortex; ST, striatum; GP, globus pallidus; TH, thalamus; CB, cerebellum.

As shown in Figure 3, intra-regional connectivity was different between P+DLB, P-DLB, and NC groups in the motor cortex (F_2, 2997_ = 199.18, *p* < 0.001, η^2^ = 0.117),the striatum (F_2, 2997_ = 3,125.38, *p* < 0.001, η^2^ = 0.676), the globus pallidus (F_2, 2997_ = 103.65, *p* < 0.001, η^2^ = 0.065), the thalamus (F_2, 2997_ = 736.23, *p* < 0.001, η^2^ = 0.337), and the cerebellum (F_2, 2997_ = 371.61, *p* < 0.001, η^2^ = 0.199). In these subdivisions, both P+DLB and P-DLB groups showed lower intra-regional connectivity than the NC group. P+DLB group showed comparable intra-regional connectivity to P-DLB in the motor cortex, striatum and globus pallidus (Figures 3A–C) but higher intra-regional connectivity in the thalamus (Figure 3D) and lower intra-regional connectivity in the cerebellum than P-DLB (Figure 3E). Intra-regional connectivities in the pons and the midbrain were comparable between the three groups.

## Discussion

In this study, patients with DLB, with or without Parkinsonism, showed lower resting metabolic connectivities between the subdivisions included in the STC circuit than the normal controls. Compared to the NC group, both P+DLB and P-DLB groups showed lower inter-regional connectivity between motor cortex–striatum, midbrain–striatum, striatum–globus pallidus, and globus pallidus–thalamus (Figures 2A,D–F), and lower intra-regional connectivity in the motor cortex, the striatum, and the globus pallidus (Figures 3A–C), indicating that the STC circuit may be disrupted in DLB, regardless of the presence of Parkinsonism. Additionally, P+DLB group showed higher connectivity between midbrain–striatum than the P-DLB group (Figure 2D) and higher motor cortex–pons, pons–cerebellum, and cerebellum–thalamus inter-regional connectivity than P-DLB and NC groups (Figures 2C,G,H), which may be compensatory hyperactivation to mitigate Parkinsonism.

These patterns of resting state brain connectivity of STC circuit and nigrostriatal pathway in DLB contrasted from those reported previously in PD. In previous resting state connectivity studies using FDG-PET and fMRI, PD showed reduced connectivity between the substantia nigra-putamen (9) and among the lower brainstem-pons-midbrain which include the substantia nigra pars compacta (30) while enhanced inter-regional connectivity between the motor cortex and the striatum (10, 11) and intra-regional connectivity in the motor cortex (33).

This suggests that the relatively preserved motor cortex may compensate the dysfunctional nigrostriatal dopaminergic pathway in PD (34). The different patterns of brain connectivity observed between DLB and PD seem to reflect the different neuropathologies between the two diseases. In DLB, Lewy pathology is prominent in the neocortex, while in the substantia nigra in PD (4, 7, 14). Considering that striatal D2 receptor upregulation was observed in PD (35) but not in DLB (36), the activation of the nigrostriatal dopaminergic projection may play a role in mitigating Parkinsonism in DLB.

This study also found that connectivity in the CTC circuit was enhanced in the P+DLB group as was in PD. In PD, compensatory CTC circuit activation was found both at rest and during motor tasks (13, 37, 38) and was positively correlated with the severity of Parkinsonism (12). The P+DLB group also showed higher motor cortex–pons, pons–cerebellum, and cerebellum–thalamus inter-regional connectivity than the P-DLB and NC groups (Figures 2C,G,H). In DLB, the CTC circuit may be activated only when Parkinsonism develops to mitigate hypokinetic symptoms as in akinesia/rigidity-type PD (12, 37).

Although the mechanism that increased connectivity of CTC circuits compensates for Parkinsonism symptoms of DLB is not known, previous studies suggest that it will play a complementary role through functional and anatomical connections of STC and CTC circuits.

In the previous PD study, putamen-motor cortex and putamen-cerebellar connectivities were increased in PD compared to normal groups, where the motor performance was negatively correlated with the connectivity of the putamen-motor cortex and positively correlated with the putamen-cerebellum (39). These results support the compensatory mechanism of the CTC circuit in PD (40). In addition, in probable DLB, the strategic binding ratio (SBR), which means the degree of dopamine active transporter (DAT) binding, has a negative correlation with the UPDRS score (41), similar to the early and mid-term PD (42). Although there is a difference in the propagation direction of Lewy pathology in DLB and PD (4, 7, 14), dopamine deposition in the striatal appears in common, supporting the argument that increased connectivity of the CTC circuits in P+DLB can compensate for dysfunction of the STC circuits.

We can also consider the effect of the anatomic connection of STC circuit and CTC circuit on motor dysfunction composition through di-synaptic connection of the cerebellum and the basal ganglia. Previous studies confirmed the presence of a disynaptic anatomical connection between the basal ganglia and the cerebellum in both animals (43) and humans (44). Abnormal neural activity in the subthalamic nucleus can be transmitted to the cerebellum *via* the pons (43). According to the “super-integrator theory,” the basal ganglia and cerebellar motor thalamus territories assimilate motivational and proprioceptive motor information previously integrated into the cortico-basal ganglia and cortico-cerebellar networks, respectively, to develop sophisticated motor signals that are transmitted in parallel pathways to cortical areas for optimal generation of motor programs (45). If this is the case, hyperactivation of the cortico-cerebellar network may mitigate Parkinsonism in DLB or PD by affecting the overall STC circuit through integration in the motor thalamus. This may be why the P+DLB group showed higher GP-TH connectivity than the P-DLB group in the current study (Figure 2F). Therefore, considering that the positive correlation with motor performance and the cerebellum of CTC circuit in the PD study (39) have various anatomical pathways, the compensation mechanism in P+DLB can be considered, and it is necessary to confirm the correlation with the motor performance of DLB in future studies.

This is the first study to see metabolic connectivity according to Parkinsonism symptoms of DLB. Therefore, it was not possible to compare the results of previous reports conducted in the same way as our study. There were several previous studies comparing the resting state connectivity between DLB and NC using FDG-PET. One have shown that the loss of metabolic connection between striatum–prefrontal cortex, striatum–sensorimotor cortex, and striatum–supplementary motor region in the DLB was more severe than in the NC (29), which is consistent with our results and shows the dysfunction of the STC motor circuit in DLB. Another metabolic connectivity study reported that the connectivity of the prefrontal region in the DLB increased compared to the NC (46). Since previous studies have reported that the prefrontal cortex plays a comprehensive role in motor system dysfunction (47–49), increased connectivity of the prefrontal cortex in DLB may be interpreted as compensating for motor dysfunction in DLB. The other study comparing the metabolic connectivity according to the stage of DLB with the normal group, DLB of early stage, shows increased inter and intra-regional connectivities in the basal ganglia and limbic system but shows decreased connectivity in the later stage (50). This suggests that the metabolic connectivity may vary depending on the severity of the disease, and it is necessary to study in the future whether the connectivity varies depending on the severity of the disease along with the motor symptom.

Resting metabolic connectivity reflects the energy consumption at the metabolic level and reflects synaptic brain metabolism, while the BOLD signal from the functional MRI reflects the ongoing neural synchronization (51). Therefore, the metabolic connectivity obtained from FDG-PET may better reflect the synaptic dysfunction of neurodegenerative diseases rather than functional connectivity obtained from fMRI. In addition, FDG-PET has better signal-to-noise ratio than fMRI (15). However, this study has several limitations to be noted. First, this study was cross-sectional, and the sample size was small. In future research, the metabolic connectivity of DLB with or without Parkinsonism with larger sample should be tested. Second, the spatial resolution of FDG-PET was not sufficient to separate brain regions into nuclear levels (52), for example, to separate the globus pallidus into an internal part and an external part. Third, the correlation between the severity of Parkinsonism and the metabolic connectivities in the STC and CTC circuits and nigrostriatal pathway was not investigated. It is also necessary to review whether the fact that the MMSE score of P-DLB was significantly higher than P+DLB did not affect the results. However, previous studies showed that there was no correlation between the motor symptom severity and the MMSE score in patients with DLB (6). Therefore, the significant difference in MMSE scores between the two groups simply indicates the stage of cognitive decline and cannot be considered to have affected the connectivity of the motor circuit according to the presence or absence of Parkinson's disease symptoms. On the other hand, since there are studies showing that the MMSE score affects the global connectivity of DLB (53), it is necessary to proceed with further research to confirm the correlation of the part related to the cognitive function, not the motor circuit.

In patients with DLB, the resting state metabolic connectivity in the STC circuit was reduced regardless of the presence of Parkinsonism. In DLB patients with Parkinsonism, the resting state metabolic connectivity was less reduced in the nigrostriatal pathway than those without Parkinsonism. DLB with Parkinsonism may possibly be differentiated from PD or PDD using the resting state metabolic connectivities of STC and nigrostriatal pathway even when non-motor symptoms appear in DLB.

## Data availability statement

The raw data supporting the conclusions of this article will be made available by the authors, without undue reservation.

## Ethics statement

All subjects were fully informed about the study and provided written informed consent to participate, obtained by either the participant or their guardians. The studies involving human participants were reviewed and approved by the Institutional Review Board of Seoul National University Bundang Hospital.

## Author contributions

EC and KWK conceptualized, designed this work, and drafted the manuscript and figure. All authors acquired and analyzed the data. All authors contributed to the article and approved the submitted version.

## Funding

This study was supported by Institute for Information & communications Technology Promotion (IITP) grant funded by the Korea government (MSIT) (2018-0-00861, Intelligent SW Technology Development for Medical Data Analysis).

## Conflict of interest

The authors declare that the research was conducted in the absence of any commercial or financial relationships that could be construed as a potential conflict of interest.

## Publisher's note

All claims expressed in this article are solely those of the authors and do not necessarily represent those of their affiliated organizations, or those of the publisher, the editors and the reviewers. Any product that may be evaluated in this article, or claim that may be made by its manufacturer, is not guaranteed or endorsed by the publisher.
